# Necrotizing Fasciitis Extending from the Left Iliopsoas to the Left Thigh Due to Retroperitoneal Penetration of the Descending Colon: A Case Report

**DOI:** 10.70352/scrj.cr.25-0195

**Published:** 2026-06-04

**Authors:** Yuhi Matsuo, Naoki Kawahara, Shu Tanizawa, Mitsuaki Kojima, Koji Morishita

**Affiliations:** Trauma and Acute Critical Care Center, Institute of Science Tokyo Hospital, Tokyo, Japan

**Keywords:** necrotizing fasciitis, retroperitoneal penetration, gastrointestinal penetration, immunosuppressed patient, steroid use

## Abstract

**INTRODUCTION:**

Necrotizing fasciitis is a severe soft tissue infection characterized by rapid destruction of the fascia and subcutaneous tissue, with a high mortality rate of 20%–40%. Although trauma and insect bites are common causes, gastrointestinal perforation as an etiology is exceedingly rare.

**CASE PRESENTATION:**

We report the case of a 70-year-old man receiving combination immunotherapy with nivolumab and ipilimumab for malignant pleural mesothelioma. The patient had been taking 12.5 mg of prednisolone daily for 6 months to manage immunotherapy-induced adrenal insufficiency and enteritis. He presented with lower abdominal pain and chills and was diagnosed with septic shock upon hospital arrival. CT revealed extensive gas extending from the left abdominal wall to the retroperitoneum and left thigh, confirming necrotizing fasciitis. Emergency surgery revealed feculent fluid near the left iliopsoas muscle, indicating retroperitoneal perforation of the descending colon. The patient underwent a left hemicolectomy and double-barrel ileostomy, followed by multiple surgical debridement and drainage procedures. After infection control, he recovered and was transferred for rehabilitation.

**CONCLUSIONS:**

Immunosuppressed patients receiving prolonged steroid therapy may be at a higher risk of developing necrotizing fasciitis due to gastrointestinal perforation. Timely infection control and aggressive surgical management are essential for improving patient outcomes.

## Abbreviations

qSOFA
: quick Sequential Organ Failure Assessment
SOFA
: Sequential Organ Failure Assessment

## INTRODUCTION

Necrotizing fasciitis is a severe soft tissue infection that rapidly destroys the fascia and subcutaneous tissue, with a reported mortality rate of 20%–40%.^1)^ It is more common in men than in women, with an average age of onset between 50 and 60 years.^2)^ Risk factors include diabetes, immunosuppressive therapy, and liver cirrhosis.^2)^ Trauma and insect bites are common causes, and delayed treatment often leads to septic shock and death.^3)^ The incidence of necrotizing fasciitis varies regionally, ranging from approximately 0.3 to 5 cases per 100000 people.^2)^ Necrotizing fasciitis secondary to gastrointestinal perforation is exceedingly rare, accounting for only a small fraction of cases.^4)^

We present a surgical case of necrotizing fasciitis extending from the retroperitoneum and iliopsoas muscle to the lower extremity due to descending colon perforation, along with a review of the relevant literature.

## CASE PRESENTATION

A 70-year-old man was undergoing combination immunotherapy with nivolumab and ipilimumab for malignant pleural mesothelioma. As a complication of immunotherapy, the patient developed enteritis and adrenal insufficiency and had been taking prednisolone (12.5 mg daily) for 6 months. The day before admission, the patient experienced lower abdominal pain and chills despite taking tramadol (25 mg). He subsequently developed left hip pain and was transported to a local hospital.

Upon arrival, the patient was hypotensive, with a systolic blood pressure of 50 mmHg despite initial fluid resuscitation. He exhibited severe tenderness and crepitus extending from the lower abdomen to the groin. Blood tests revealed elevated inflammatory markers, including a white blood cell count of 10700/μL and a C-reactive protein level of 1.61 mg/dL. His serum lactate level was 3.1 mmol/L, and norepinephrine (0.03 μg/kg/min) was required to maintain a mean arterial pressure ≥65 mmHg. He had a qSOFA score of 2 and a SOFA score of 3 at admission. Despite adequate fluid resuscitation, his blood pressure showed minimal improvement, and his lactate level remained ≥3 mmol/L, confirming the diagnosis of septic shock.

**Fig. 1:**
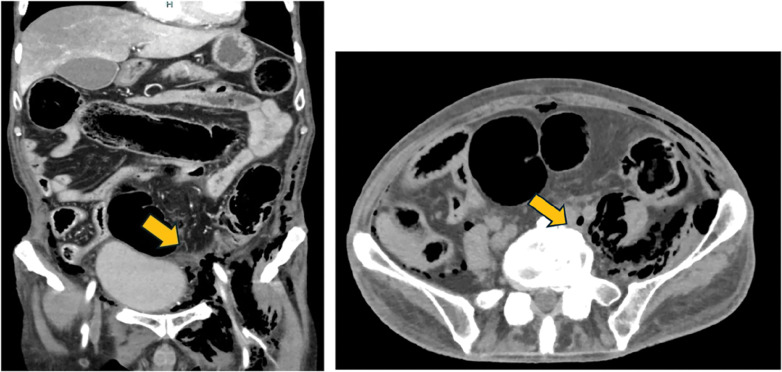
CT scan from the referring hospital. The yellow arrows indicate an abscess in the abdominal cavity.

A CT scan revealed extensive gas extending from the left abdominal wall and retroperitoneum to the left thigh muscles and anterior pubic region, without free air or ascites in the abdominal cavity (**Fig. 1**). Based on these findings, the patient was diagnosed with necrotizing fasciitis and septic shock and was transferred to our hospital for emergency surgery.

**Fig. 2:**
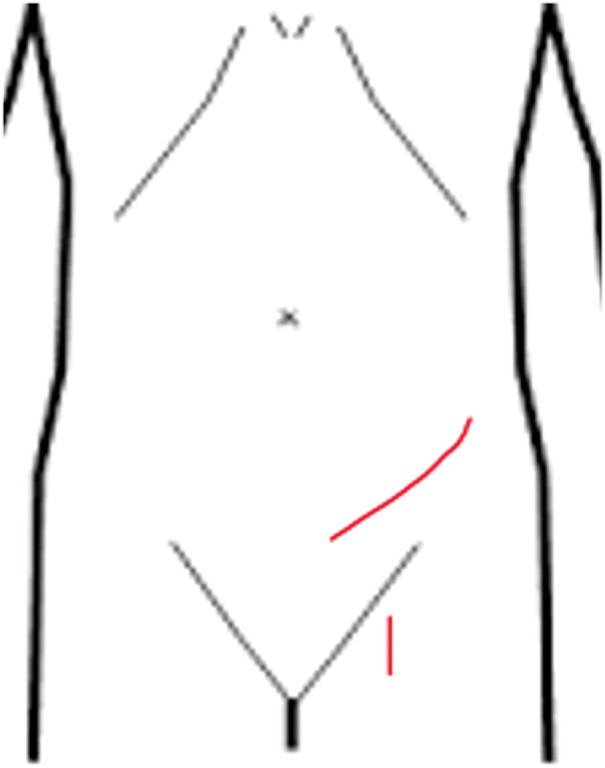
Schematic illustration. The red lines represent the planned surgical incision.

**Fig. 3:**
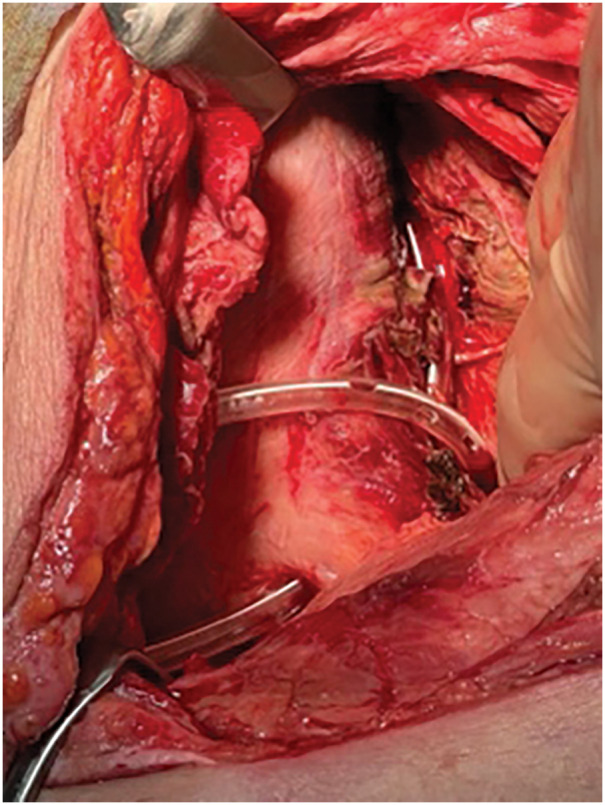
Wound appearance immediately after initial debridement on POD 1.

Empirical antibiotic therapy was initiated, and incision and drainage were performed for infection control (**Figs. 2** and **3**). A retroperitoneal approach via a left abdominal oblique incision revealed feculent fluid near the left iliopsoas muscle, indicating retroperitoneal penetration of the gastrointestinal tract. Laparotomy revealed severe inflammation and dense adhesions in the descending colon, consistent with retroperitoneal penetration. The adhesions between the descending colon and retroperitoneum were carefully dissected, followed by a left hemicolectomy with double-barrel ileostomy. Although the patient initially presented with shock, vital stability during surgery made Hartmann’s procedure a safe alternative; however, the intraoperative hemodynamic condition stabilized with a relatively low dose of norepinephrine. The bowel condition was favorable, and primary anastomosis was technically feasible. Ileostomy was selected instead of constructing a stoma in the inflamed colon, as it allows for a safer and less technically demanding stoma closure.

We therefore considered that a 1-stage anastomosis would not significantly prolong the operative time or increase the surgical burden. Given the patient’s immunocompromised status and impaired wound healing capacity, we constructed a proximal ileostomy to minimize fecal load and pressure on the anastomosis, thereby reducing the risk of leakage. In addition, incision and drainage were performed for the infected area in the left thigh.

**Fig. 4:**
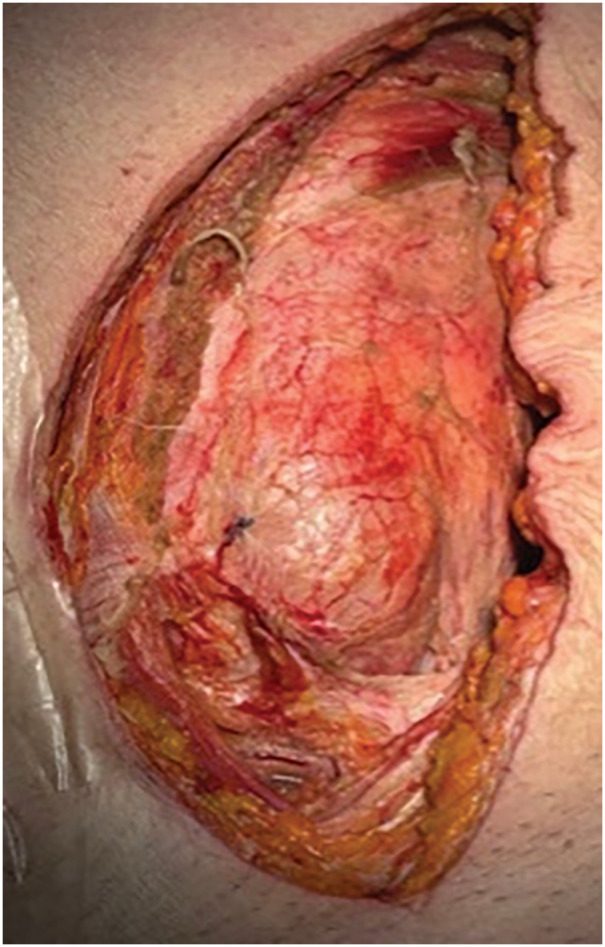
Wound condition on POD 5 during the healing period.

Vasopressors were discontinued on POD 2. Hyperbaric oxygen therapy was initiated on POD 5 (**Fig. 4**) to enhance wound healing and exert antibacterial effects. On POD 7, additional drains were inserted in the retroperitoneum and left thigh for continuous irrigation, and negative pressure wound therapy was initiated for the left abdominal wall. A CT scan on POD 9 confirmed the absence of intra-abdominal abscesses, and the pelvic drain was removed.

**Fig. 5:**
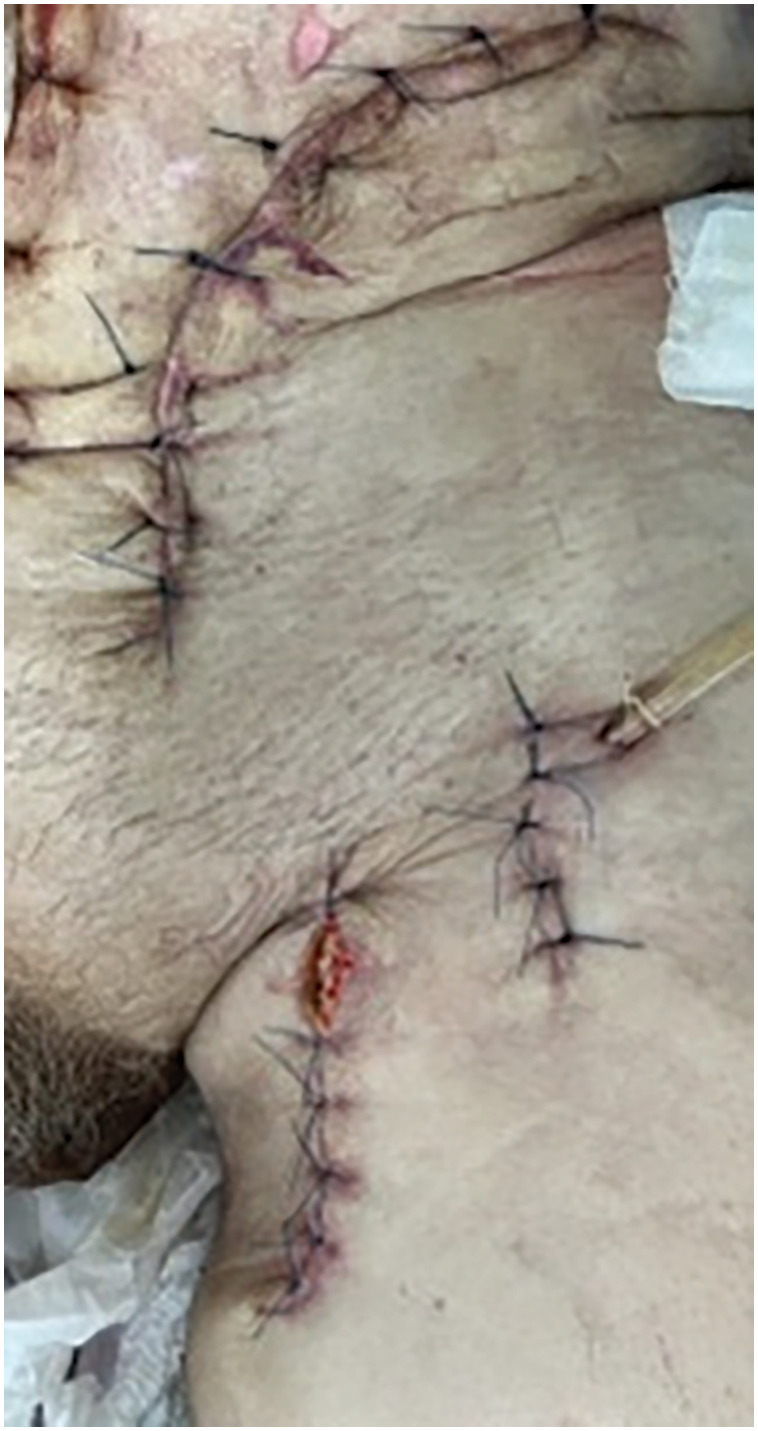
Wound condition on POD 20 during the healing period.

**Fig. 6:**
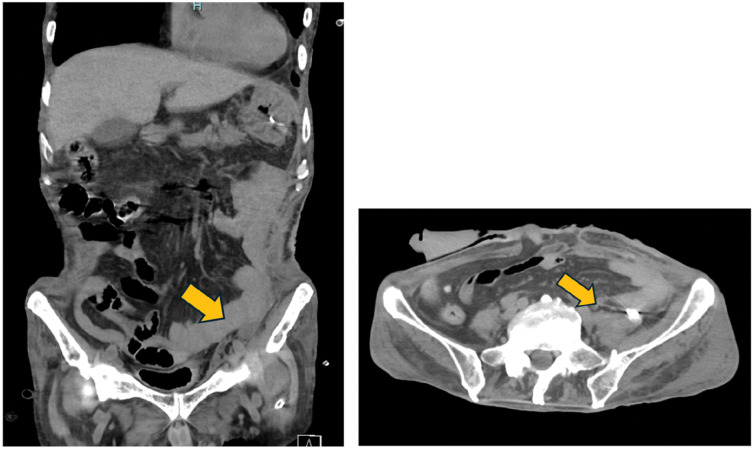
CT scan on POD 30. The yellow arrows show resolution of the abscesses in the abdominal cavity.

On POD 14, the patient developed a sudden fever of 38°C, accompanied by rising inflammatory markers. A CT scan on POD 16 revealed an anastomotic leak in the colon, necessitating additional resection, laparotomy with drainage, and the formation of a transverse colostomy with additional drains placed under the left diaphragm. During the 2nd operation, the preexisting ileostomy was left in place without being closed. A transverse colostomy was constructed as a diverting stoma to prioritize sepsis control. Considering the patient’s unstable condition and the complexity of the clinical course at that time, the ileostomy was temporarily left in place, with plans for possible closure once the patient’s condition stabilized. The left subdiaphragmatic and pelvic drains were removed on POD 26 (**Fig. 5**). A CT scan on POD 30 confirmed resolution of the abscesses (**Fig. 6**), allowing the removal of the retroperitoneal drain. The left thigh drain was removed, and the wound was closed on POD 34. The patient was transferred to a rehabilitation facility on POD 40.

**Fig. 7:**
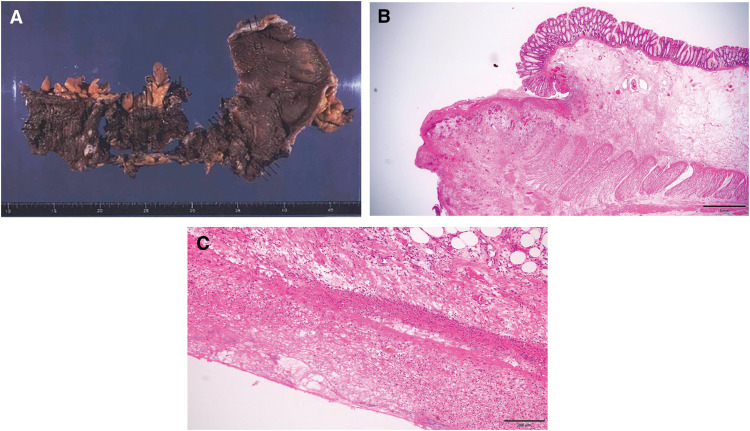
Histopathological findings of the resected colon. (**A**) Macroscopic appearance of the resected colon. The specimen shows a segment of the colon with marked wall thickening, discoloration, and tissue fragility. The suspected site of perforation is identified on the anal side, with surrounding inflammatory changes and fibrinous exudate. (**B**) Low-power view of H&E-stained section showing transmural necrosis and disruption of the bowel wall architecture at the site of perforation. (**C**) High-power view demonstrating dense neutrophilic infiltration, fibrin deposition, and necrotic changes extending into adjacent adipose tissue. No evidence of malignancy or diverticular disease is identified. Scale bar = 1 mm (**B**) and 200 μm (**C**). H&E, hematoxylin and eosin

Histopathological examination of the resected colon revealed transmural necrosis with dense neutrophilic infiltration and fibrin deposition at the site of perforation. Inflammatory changes extended into the surrounding adipose tissue. No evidence of malignancy, diverticular disease, or any specific pathological cause of perforation was identified (**Fig. 7**).

## DISCUSSION

This case highlights a rare presentation of necrotizing fasciitis secondary to retroperitoneal perforation of the descending colon in a patient undergoing immunotherapy with nivolumab and ipilimumab, along with long-term steroid use. A PubMed search for “retroperitoneal (perforation OR penetration) AND necrotizing fasciitis” identified 34 reported cases.^5–38)^ At least 12 patients (35%) succumbed to septic multi-organ failure,^6,12,14,18–22,26,27,33,34)^ underscoring the high mortality associated with necrotizing fasciitis originating from gastrointestinal penetration (**Table 1**).

**Table 1:** Summary of reported cases of necrotizing fasciitis originating from gastrointestinal penetration

Authors (publication year)	Patient demographic (age/sex)	Underlying diseases	History of steroid or chemotherapy treatment	Treatment	Outcome
Beg et al. (2020)^5)^	42/Male	Appendicitis	—	Debridement, drainage	Survived
Tarekegn et al. (2024)^6)^	19/Male	Appendicitis	—	Right hemicolectomy, debridement, drainage	Dead
Kobayashi et al. (2023)^7)^	72/Male	Descending colon cancer	—	Transverse colostomy, debridement, drainage	Survived
Roje et al. (2011)^8)^	56/Male	Perforation of cecum, necrosis of ascending colon	—	Debridement, fasciectomy, right orchiectomy, right hemicolectomy, diverting colostomy in the descending colon	Survived
Tee et al. (2020)^9)^	70/Male	Non-specified, caused by toothpick	—	Drainage, debridement	Survived
Smith and McKenney (2016)^10)^	Unknown	Sigmoid diverticulitis	—	Unknown	Non-specified
Benesch and Bussey (2020)^11)^	61/Female	Sigmoid diverticulitis	—	Left hemicolectomy, debridement, drainage, end colostomy creation	Survived
Khefacha et al. (2023)^12)^	67/Male	Secondary aortoduodenal fistula	—	Debridement, resection of perforated bowel between the 3rd duodenum and the first jejunal loop, prosthetic explanation	Dead
Kousoulas et al. (2018)^13)^	25/Female	Renal rupture	—	Right nephrectomy, right hemicolectomy, debridement, right thigh amputation	Survived
Chohan et al. (2010)^14)^	Unknown/Female	Cecum carcinoma	—	Non-specified	Dead
Bouassida et al. (2015)^15)^	38/Male	Cecal diverticulitis	—	Right hemicolectomy, debridement, ileocolostomy, drainage	Survived
Celik and Senocak (2021)^16)^	68/Female	Rectosigmoid tumor	Chemotherapy	Hartmann’s procedure	Survived
Vervaecke et al. (2022)^17)^	62/Male	WON	—	—	Unknown
Markou et al. (2005)^18)^	70/Female	Colonic perforation	Steroid	Hemicolectomy, debridement, Hartmann’s diverting colostomy	Dead
Kanadashvili et al. (2021)^19)^	78/Male	Appendicitis	—	Laparoscopic appendectomy, debridement, right thigh amputation	Dead
Hua et al. (2015)^20)^	50/Male	Perforated appendicitis	—	Appendectomy, debridement, drainage	Dead
Michalopoulos et al. (2013)^21)^	60/Male	Retroperitoneal stromal cell tumor	—	Left hemicolectomy, transverse colostomy creation surgery, debridement	Dead
Heidelberg et al. (2020)^22)^	84/Male	Perforated cecal cancer	—	Only antibiotics	Dead
Wilharm et al. (2010)^23)^	21/Male	Appendicitis	—	Appendectomy, debridement	Survived
Elahabadi et al. (2021)^24)^	25/Male	Appendicitis	—	Debridement	Survived
Mammadli et al. (2017)^25)^	52/Male	Colonic cancer	—	Right hemicolectomy, colostomy, pulmonary segmentectomy	Survived
Pouriki et al. (2017)^26)^	82/Male	Cecal adenocarcinoma, diabetes	—	Right hemicolectomy, colostomy, debridement	Dead
Taif and Alrawi (2014)^27)^	26/Female	Appendicitis	—	Debridement	Dead
Fu et al. (2009)^28)^	73/Male	Rectal tumor	Chemotherapy, radiation	Dysfunctional transverse loop colostomy, debridement	Survived
Fukui et al. (2018)^29)^	85/Male	Sacral pressure ulcer	—	Debridement	Survived
Underwood et al. (2008)^30)^	51/Male	Sigmoid colonic diverticulitis	—	Hartmann’s procedure, debridement, drainage	Survived
Wiberg et al. (2012)^31)^	57/Male	Sigmoid colonic perforation	Steroid	Hartman’s procedure, debridement	Survived
Marron et al. (2006)^32)^	52/Male	Cecum carcinoma	—	Debridement, right hemicolectomy, cecum resection	Survived
Papanikolas et al. (2020)^33)^	70/Male	Appendicitis	—	Ileocolic resection, debridement, amputation of the right leg	Dead
Lam et al. (1996)^34)^	Unknown	Sigmoid colon tumor	—	Unknown	Dead
Coulier et al. (2012)^35)^	41/Male	Appendicitis	—	Debridement, appendectomy	Survived
	55/Female	Sigmoid colon diverticulitis	—	Hartman’s procedure	Survived
Lee et al. (2003)^36)^	Unknown	Perforated duodenum	—	Unknown	Unknown
Mazza et al. (1987)^37)^	Unknown	Appendicitis	—	Unknown	Unknown
Klutke et al. (1988)^38)^	Unknown	Sigmoid colon diverticulitis	—	Sigmoid colectomy, debridement	Unknown

WON, walled-off necrosis

The average patient age was 55.6 years, and only 1 patient (3%) had a history of diabetes.^26)^ The most common cause was appendicitis or cecal perforation (16 cases, 47%),^5,6,8,14,15,19–24,26,27,32,33,35,37)^ followed by tumor-related perforation (9 cases, 26%)^7,14,21,22,25,26,28,32,34)^ and diverticulitis (6 cases, 18%).^10,11,15,30,35,38)^ A history of steroid use was reported in only 2 cases (6%),^18,31)^ although gastrointestinal perforation is known to occur more frequently in patients receiving steroids.^39)^ In addition, 2 cases (6%) were associated with chemotherapy.^16,28)^ Nivolumab plus ipilimumab has been reported to cause enteritis in 4%–8% of cases.^40)^ In this case, the colon showed severe adhesions and inflammation, which may have been associated with immunotherapy-induced enteritis. Although no distinct lesions were identified, immunosuppression from immunotherapy and steroid use may have contributed to bowel wall fragility, leading to perforation and subsequent infection. The anti-inflammatory effects of steroids may have further delayed symptom onset, allowing the progression to necrotizing fasciitis.

Initially, gastrointestinal perforation was not suspected. However, the intraoperative detection of feculent fluid allowed for early diagnosis and timely surgical intervention, including colectomy and stoma formation, which were crucial for infection control.

Retroperitoneal colonic perforation should be suspected as a potential source of necrotizing fasciitis in immunocompromised patients, and prompt surgical management is crucial.

## CONCLUSIONS

Patients receiving immunotherapy, prolonged steroid therapy, or treatment for chronic enteritis may have an increased risk of gastrointestinal wall compromise, predisposing them to retroperitoneal perforation and subsequent necrotizing fasciitis. Although rare, such cases require early recognition, prompt source control, and aggressive surgical intervention to improve patient outcomes.
